# Supplementary material for: Exploring the Distinct Binding and Activation Mechanisms for Different CagA Oncoproteins and SHP2 by Molecular Dynamics Simulations
Source: Molecules. 2021 Feb 5;26(4):837. doi: 10.3390/molecules26040837 (PMC7916045; doi:10.3390/molecules26040837)
Supplement: Supplementary file 1 [file molecules-26-00837-s001.pdf]

Supplementary Material

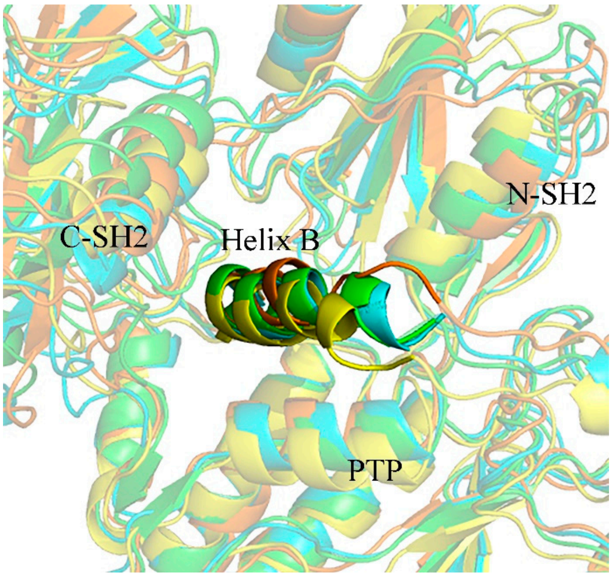

Figure S1. The superimposed structure of helix B for system SHP2 (yellow), system EPIYA−D−N (blue), system EPIYA−C−N (orange), and system EPIYA−C−DUAL (green).

Table S1. Decomposition of binding energy (kcal mol⁻¹) of key residues for system EPIYA−D−N.

Residues	ΔG_{vdW}	ΔG_{ele}	ΔG_{GB}	ΔG_{SA}	ΔG_{bind}
ARG32	0.72 ± 0.96	−102.31 ± 2.69	87.97 ± 2.00	−0.10 ± 0.02	−13.71 ± 1.13
LYS55	−3.38 ± 0.90	−118.00 ± 3.95	109.82 ± 3.63	−0.57 ± 0.04	−12.12 ± 2.08
SER34	0.49 ± 0.92	−22.17 ± 1.62	15.16 ± 0.90	−0.01 ± 0.00	−6.52 ± 1.02
SER36	0.06 ± 0.92	−20.69 ± 1.83	14.78 ± 1.18	−0.29 ± 0.06	−6.25 ± 0.93
LYS35	−0.70 ± 0.47	−69.38 ± 3.03	65.21 ± 2.94	−0.04 ± 0.01	−4.90 ± 0.65
LYS91	−3.47 ± 0.75	−74.04 ± 5.74	75.28 ± 6.31	−0.70 ± 0.12	−2.94 ± 1.17
ILE54	−1.83 ± 0.36	−0.33 ± 0.36	0.62 ± 0.27	−0.20 ± 0.03	−2.79 ± 0.42
TYR66	−3.06 ± 0.65	1.90 ± 1.79	−0.84 ± 1.59	−0.57 ± 0.10	−2.57 ± 0.66
GLY68	−1.62 ± 0.39	−2.37 ± 2.61	2.00 ± 2.27	−0.37 ± 0.04	−2.36 ± 0.60
THR42	−0.16 ± 0.76	−8.23 ± 1.94	6.18 ± 1.07	−0.04 ± 0.01	−2.25 ± 0.83

Table S2. Decomposition of binding energy (kcal mol⁻¹) of key residues for system EPIYA−C−N.

Residues	ΔG_{vdW}	ΔG_{ele}	ΔG_{GB}	ΔG_{SA}	ΔG_{bind}
ARG32	0.72 ± 0.92	−103.77 ± 6.30	89.48 ± 4.40	−0.08 ± 0.02	−13.64 ± 2.30
LYS55	−2.22 ± 0.70	−114.16 ± 8.99	107.61 ± 6.95	−0.34 ± 0.05	−9.12 ± 3.19
LYS35	−0.74 ± 0.68	−83.88 ± 17.93	78.39 ± 16.55	−0.13 ± 0.13	−6.35 ± 1.83
SER34	0.23 ± 0.88	−20.05 ± 1.95	13.70 ± 1.14	−0.00 ± 0.00	−6.12 ± 1.17
SER36	0.11 ± 1.07	−19.85 ± 2.52	14.22 ± 1.50	−0.02 ± 0.11	−5.72 ± 1.40
LYS89	−2.00 ± 0.77	−103.17 ± 20.10	101.03 ± 18.34	−0.54 ± 0.11	−4.67 ± 2.14
HIS53	−2.98 ± 0.75	−2.08 ± 1.34	1.40 ± 1.04	−0.50 ± 0.07	−4.16 ± 1.10
LYS91	−3.20 ± 0.77	−96.98 ± 19.18	96.89 ± 18.25	−0.71 ± 0.13	−4.00 ± 1.62
THR42	−0.21 ± 0.82	−7.54 ± 4.20	5.37 ± 2.89	−0.04 ± 0.02	−2.42 ± 1.30

Table S3. Decomposition of binding energy (kcal mol[−1]) of key residues for system EPIYA−D−C.

Residues	ΔG_{vdW}	ΔG_{ele}	ΔG_{GB}	ΔG_{SA}	ΔG_{bind}
ARG138	0.49 ± 0.91	−105.64 ± 2.39	92.47 ± 1.92	−0.09 ± 0.02	−12.77 ± 1.68
GLN141	−0.54 ± 0.78	−22.79 ± 7.23	14.17 ± 3.98	−0.16 ± 0.09	−9.32 ± 3.68
ARG186	0.66 ± 0.87	−105.47 ± 3.27	96.41 ± 2.07	−0.17 ± 0.02	−8.57 ± 1.12
SER140	0.13 ± 0.85	−20.33 ± 1.93	13.52 ± 1.00	−0.02 ± 0.02	−6.70 ± 1.33
LYS120	−2.56 ± 0.93	−87.66 ± 8.56	88.30 ± 7.25	−0.87 ± 0.06	−4.79 ± 1.84
HIS169	−2.97 ± 0.59	−1.34 ± 1.00	0.36 ± 0.88	−0.47 ± 0.03	−4.41 ± 0.59
VAL203	−2.60 ± 0.43	−0.77 ± 0.59	0.26 ± 0.37	−0.46 ± 0.04	−3.57 ± 0.56
THR205	−2.60 ± 0.46	−3.90 ± 0.92	3.87 ± 0.97	−0.54 ± 0.04	−3.17 ± 0.57
GLU204	−2.80 ± 0.44	57.71 ± 1.95	−57.59 ± 1.96	−0.27 ± 0.03	−2.95 ± 0.52
VAL170	−2.30 ± 0.35	−1.78 ± 0.40	1.56 ± 0.30	−0.20 ± 0.03	−2.72 ± 0.45
VAL148	−1.42 ± 0.24	−2.39 ± 0.40	1.34 ± 0.36	−0.08 ± 0.01	−2.55 ± 0.30
GLY183	−1.82 ± 0.32	−8.11 ± 0.83	7.54 ± 0.56	−0.15 ± 0.02	−2.54 ± 0.60
GLY184	−0.93 ± 0.45	−0.98 ± 1.40	−0.36 ± 0.78	−0.22 ± 0.03	−2.50 ± 0.64

Table S4. Decomposition of binding energy (kcal mol[−1]) of key residues for system EPIYA−C−C.

Residues	ΔG_{vdW}	ΔG_{ele}	ΔG_{GB}	ΔG_{SA}	ΔG_{bind}
ARG138	0.64 ± 0.92	−105.56 ± 3.07	91.75 ± 2.45	−0.10 ± 0.02	−13.27 ± 1.56
GLN141	−0.84 ± 0.53	−13.63 ± 7.17	8.41 ± 5.45	−0.09 ± 0.05	−6.15 ± 2.29
SER142	0.12 ± 0.88	−20.02 ± 1.82	13.99 ± 1.00	−0.16 ± 0.02	−6.07 ± 1.21
SER140	0.21 ± 0.84	−19.54 ± 2.44	13.31 ± 1.16	−0.01 ± 0.01	−6.02 ± 1.40
HIS169	−2.94 ± 0.56	−1.28 ± 1.00	0.13 ± 0.79	−0.48 ± 0.03	−4.57 ± 0.65
LYS120	−2.49 ± 0.98	−85.43 ± 8.90	84.35 ± 7.70	−0.86 ± 0.10	−4.43 ± 1.90
VAL170	−2.55 ± 0.36	−1.91 ± 0.44	−1.71 ± 0.32	−0.27 ± 0.03	−3.01 ± 0.49
VAL203	−1.87 ± 0.59	−0.50 ± 1.04	−0.06 ± 0.66	−0.34 ± 0.06	−2.76 ± 0.86
GLU204	−2.83 ± 0.37	60.29 ± 2.33	−59.87 ± 2.33	0.25 ± 0.03	−2.66 ± 0.53
VAL148	−1.28 ± 0.37	−2.49 ± 0.41	−1.49 ± 0.34	−0.08 ± 0.02	−2.35 ± 0.54
THR205	−2.37 ± 0.59	−2.91 ± 1.43	3.56 ± 1.18	−0.50 ± 0.06	−2.23 ± 1.26

Table S5. Decomposition of binding energy (kcal mol[−1]) of key residues for system DUAL−C (N−SH2).

Residues	ΔG_{vdW}	ΔG_{ele}	ΔG_{GB}	ΔG_{SA}	ΔG_{bind}
ARG32	0.59 ± 0.91	−127.89 ± 3.42	112.86 ± 2.73	−0.07 ± 0.02	−14.51 ± 1.63
LYS55	−1.63 ± 0.73	−134.06 ± 11.59	126.21 ± 8.80	−0.26 ± 0.05	−9.74 ± 4.03
SER34	0.33 ± 0.87	−19.17 ± 1.93	12.81 ± 1.23	−0.00 ± 0.00	−6.03 ± 1.12
LYS35	−0.44 ± 0.57	−104.83 ± 13.35	99.80 ± 12.52	−0.10 ± 0.12	−5.56 ± 1.31
HIS53	−4.07 ± 0.70	−1.57 ± 1.63	0.98 ± 1.15	−0.50 ± 0.04	−5.17 ± 0.84
LYS91	−3.37 ± 0.88	−121.37 ± 12.68	120.42 ± 11.44	−0.76 ± 0.08	−4.98 ± 1.67
SER36	0.32 ± 0.86	−18.78 ± 2.13	14.38 ± 1.22	−0.09 ± 0.04	−4.17 ± 1.23
THR42	−0.30 ± 0.73	−9.36 ± 2.88	6.63 ± 1.87	−0.02 ± 0.02	−3.05 ± 1.10
LYS89	−1.69 ± 0.78	−89.80 ± 16.48	89.47 ± 14.65	−0.32 ± 0.14	−2.33 ± 2.46

Table S6. Decomposition of binding energy (kcal mol^{-1}) of key residues for system DUAL−C (C−SH2).

Residues	ΔG_{vdW}	ΔG_{ele}	ΔG_{GB}	ΔG_{SA}	ΔG_{bind}
ARG138	0.37 ± 0.95	−126.23 ± 4.36	112.53 ± 3.55	−0.13 ± 0.02	−13.45 ± 1.82
SER142	0.32 ± 0.94	−19.44 ± 1.94	13.68 ± 1.40	−0.17 ± 0.04	−5.62 ± 1.24
GLN141	−0.44 ± 0.59	−8.44 ± 3.87	4.25 ± 3.36	−0.03 ± 0.03	−4.66 ± 0.96
HIS169	−2.89 ± 0.57	0.60 ± 1.06	−1.46 ± 0.92	−0.48 ± 0.03	−4.22 ± 0.60
SER140	−0.75 ± 0.66	−15.78 ± 2.47	12.46 ± 1.20	−0.03 ± 0.01	−4.10 ± 1.48
THR205	−2.63 ± 0.56	−3.84 ± 1.19	3.92 ± 1.26	−0.54 ± 0.04	−3.10 ± 0.66
GLU204	−2.81 ± 0.43	74.68 ± 2.66	−74.53 ± 2.60	−0.26 ± 0.03	−2.91 ± 0.52
LYS120	−3.23 ± 0.63	−77.64 ± 17.49	78.87 ± 16.66	−0.58 ± 0.10	−2.60 ± 1.27
VAL170	−2.20 ± 0.34	−1.56 ± 0.44	−1.46 ± 0.36	−0.20 ± 0.03	−2.50 ± 0.45
VAL203	−1.93 ± 0.49	0.22 ± 1.16	−0.36 ± 0.75	−0.35 ± 0.06	−2.42 ± 0.71

Table S7. Properties of H−bonds between SHP2 and EPIYA in systems EPIYA−D−N and EPIYA−C−N.

Systems	Acceptor ~ Donor	Occupied (%)
EPIYA−D−N	A1@H~HIS53@O	75
	T2@O~LYS91@H	62
	PTR0@O3P~ARG32@H	60
	PTR0@O1P~SER34@H	58
	PTR0@O1P ~SER36@H	54
	PTR0@O1P~THR42@H	52
	PTR0@O3P~LYS35@H	48
	A1@O~LYS55@H	44
	A−6@O~TYR66H	37
	D4@H~LYS89@O	35
	D4@O~GLY68@H	34
EPIYA−C−N	A1@H~HIS53@O	70
	T2@O~LYS91@H	61
	D4@O~GLY68@H	54
	PTR0@O2P~SER36@H	45
	PTR0@O1P~THR42@H	44
	PTR0@O2P~SER34@H	43
	PTR0@O1P~ARG32@H	42
	D4@H~LYS89@O	41

Table S8. Properties of H−bonds between SHP2 and EPIYA in system EPIYA−D−C and EPIYA−C−C.

Systems	Acceptor ~ Donor	Occupied (%)
EPIYA−D−C	A1@H~HIS169@O	80
	PTR0@O2P~ARG138@H	75
	D4@H~VAL203@O	69
	PTR0@O1P~SER142@OG	63
	D6@O~ARG186@H	62
	T2@O~THR205@H	61
	PTR0@O1P~SER140@H	57
	D6@O~GLY184@H	47
	PTR0@O3P~GLN141@H	37
EPIYA−C−C	A1@H~HIS169@O	84
	T2@O~THR205@H	56
	PTR0@O2P~ARG138@H	55
	PTR0@O1P~SER142@H	44
	PTR0@O1P~SER140@OG	43
	PTR0@O3P~GLN141@H	33

Table S9. Properties of key H−bonds in system SHP2.

Key Regions	Acceptor ~ Donor	Occupied (%)
N−SH2−PTP	ASN58@H~GLN506@ND2	86
	GLY60@O~ GLN510@HE	85
	ALA72@O~GLN506@HE	64
	THR73@HG~GLU258@OE2	50
	GLU76@OE1~ARG265@H	49
	ASP61@OD1~ARG465@H	46
Helix B	GLU250@OE2~ ARG111@H	91
	GLN255@OE1~ARG498@H	86
	GLU252@OE1~ARG4@H	73
	LYS244@H~HIS114@O	70
	GLU258@OE2~THR73@H	50
	LYS242@O~HIS116@H	50
	LYS35@H~GLU249@OE2	40

Table S10. Properties of key H−bonds in system EPIYA−D−N.

Key Regions	Acceptor ~ Donor	Occupied (%)
N−SH2−PTP	ALA72@O~GLN506@HE	95
	ASN58@H~GLN506@OE1	90
	GLY60@O~GLN510@HE	77
	ASP61@OD1~ALA461@H	77
	ASP61@OD2~GLY464@H	74
Helix B	GLU258@OE1~ARG498@H	89
	GLN255@OE1~ARG498@HG	87
	GLU258@OE1~ARG4@H	87
	GLN256@O~SER3@HG	79
	GLN256@OE1~ARG4@HE	64
	GLU250@OE1~ARG111@H	50
	GLU252@OE2~ARG4@H	45
	TYR63@OH~GLN255@HE	31

Table S11. Properties of key H−bonds in system EPIYA−C−N.

Key Regions	Acceptor ~ Donor	Occupied (%)
N−SH2−PTP	ALA72@O~GLN506@H	93
	ASN58@H~GLN506@OE1	87
	GLY60@O~GIN510@H	85
	GLU76@OE1~SER502H	63
	ASP61@OD2~ARG465@H	60
Helix B	GLU252@OE1~ARG4@H	92
	GLN256@O~ARG4@H	81
	GLN257@H~SER109@O	75
	GLN256@H~PHE7@O	67
	GLN255@H~TYR63@OH	52
	LEU262@H~GLN79@OE1	42

Table S12. Properties of key H−bonds in system EPIYA−DUAL.

Key Regions	Acceptor ~ Donor	Occupied (%)
N−SH2−PTP	ASP61@OD2~ALA461@H	94
	ALA72@O~GLN506@H	86
	ASN58@H~GLN506@OE1	85
	GLY60@O~GLN510@H	81
	ASP 61@OD1~GLY464@H	77
	ASP61@OD1~ILE463@H	73
	ASP61@OD2~GLY462@H	44
Helix B	GLN256@O~SER3@H	95
	GLU252@OE1~ARG4@H	90
	GLN255@OE1~ARG498@H	80
	GLN256@OE1~ARG4@H	73
	GLU258@OE1~ARG498@H	54
	GLU250@OE2~ARG111@H	49
	LYS244@H~THR108@O	43
